# Effect of Combined Pulley and Vibration Therapy on Spasticity and Motor Function in Children with Cerebral Palsy and Genu Recurvatum: A Quasi-Experimental Study

**DOI:** 10.7759/cureus.111976

**Published:** 2026-07-03

**Authors:** Shrushti R Sonmale, Mandar Malawade

**Affiliations:** 1 Department of Pediatric Neurosciences Physiotherapy, Krishna College of Physiotherapy, Krishna Vishwa Vidyapeeth (Deemed to be University), Karad, IND

**Keywords:** cerebral palsy, genu recurvatum, pediatric neurorehabilitation, pulley therapy, spasticity, vibration therapy

## Abstract

Introduction

Cerebral palsy is a non-progressive neurological disorder affecting movement, posture, and muscle tone. Spasticity commonly leads to secondary musculoskeletal deformities such as genu recurvatum, which causes knee hyperextension during gait and negatively affects balance, posture, and functional mobility. Conventional rehabilitation often shows limited effectiveness in simultaneously improving motor function and reducing spasticity. Pulley therapy promotes task-oriented strengthening and motor control, while vibration therapy may reduce spasticity through neuromodulatory effects and improved proprioception. The combination of these therapies in children with genu recurvatum and cerebral palsy, however, is not well documented by research.

Methodology

A quasi-experimental, single-group pre-post interventional study was conducted at the Department of Pediatric Physiotherapy, Krishna College of Physiotherapy, Krishna Vishwa Vidyapeeth, Karad, India. It is acknowledged that the single-group pre-post design limits causal inference; observed improvements may be attributable in part to factors such as natural maturation, concurrent standard physiotherapy, therapist attention, or repeated testing effects rather than exclusively to the combined intervention. Fifteen children diagnosed with spastic cerebral palsy and genu recurvatum, aged between five and 12 years, were recruited through convenience sampling and categorised under Gross Motor Function Classification System (GMFCS) Levels I and III. Eight participants were classified as GMFCS Level I and seven participants as GMFCS Level III. Participants received a multicomponent intervention comprising standard physiotherapy, pulley therapy, and vibration therapy. As all components were delivered simultaneously, the independent contribution of each component could not be isolated.

Results

Children with spastic cerebral palsy and genu recurvatum showed statistically significant improvements in gross motor function and decreases in spasticity, according to the combined post-intervention analysis. Gross Motor Function Measure-88 (GMFM-88) scores (dimensionless proportion scores ranging from 0 to 1) improved from 0.690 ± 0.05 to 0.762 ± 0.06, Modified Ashworth Scale (MAS) scores reduced from 1.90 ± 0.38 to 1.40 ± 0.35, and knee hyperextension decreased from 11.00° ± 1.8 to 7.80° ± 1.5 following the 12-week intervention program (p < 0.001).

Conclusion

In children with cerebral palsy with genu recurvatum, pulley therapy in conjunction with vibration therapy was associated with significant improvements in motor function and spasticity, showing preliminary promise as a rehabilitation strategy for this population. The integrated protocol may help enhance neuromuscular control, functional mobility, and overall physical performance in this population.

## Introduction

Cerebral palsy is a non-progressive neurodevelopmental condition caused by injury to the developing brain during the fetal period, infancy, or early childhood, resulting in persistent deficits in mobility, posture, and motor and cognitive development, with secondary complications that may worsen progressively if left untreated [1,2].

The central nervous system's typical motor control mechanisms are disrupted in the pathophysiology of cerebral palsy, which results in abnormal muscle tone, poor selective motor control, and postural dysfunction [3]. In addition to severely limiting functional capacities and lowering quality of life, spasticity also contributes to the development of pain, contractures, and subsequent orthopedic abnormalities [4].

Among the various musculoskeletal complications that can accompany cerebral palsy, including crouched gait, scoliosis, and flexion contractures of the upper extremities, genu recurvatum is a clinically significant presentation characterized by severe hyperextension of the knee, which raises the likelihood of degenerative knee joint changes, compromises stance phase stability, and increases energy consumption during walking [5]. Multiple factors contribute to its development, including weak knee flexors, adaptive shortening of plantar flexor muscles, and chronic plantar flexor spasticity that generates an extension moment at the knee during standing [6].

Comprehensive intervention approaches targeting both the neurophysiological deficits and their functional ramifications are necessary for the comprehensive therapy of spasticity and its related problems. Injections of botulinum toxin type A, oral anti-spasticity drugs, physical therapy, orthotic management, and surgical approaches are presently recognized as conventional treatment methods. Nonetheless, recent investigations indicate that pharmaceutical and rehabilitative interventions, when combined, yield more effective results than single-modality therapies [3, 4, 7].

Pulley therapy is a novel treatment strategy that promotes functional movement patterns while facilitating active-assistive and resistive exercises using pulley-based resistance devices [8]. The technique allows for task-specific training that targets the muscular weakness and selective motor control deficiencies frequently seen in CP [9]. Systems for pulley therapy offer carefully calibrated resistance that may be adjusted to fit the child's existing functional ability, enabling gradual strengthening without making spasticity worse [10].

In the context of outcome measurement, the Modified Ashworth Scale (MAS) and Gross Motor Function Measure (GMFM) are proven, trustworthy tools for evaluating spasticity and physical motor function in children with cerebral palsy. The GMFM-88 offers a thorough assessment of overall motor abilities in the areas of sitting, standing, crawling and kneeling activities, lying and rolling, walking, running tasks, and leaping. Furthermore, functional mobility measures, kinematic analysis, and gait velocity measurement offer objective assessment of therapy results [5,9,11].

By methodically assessing the combined benefits of pulley treatment and vibration therapy, particularly in children with genu recurvatum, this study fills a significant vacuum in the literature on pediatric cerebral palsy rehabilitation. While separate investigations have revealed the effectiveness of vibration treatment and pulley-based training, no published study has looked at their combined use in pediatric patients with cerebral palsy who present with this particular orthopedic abnormality.

## Materials and methods

The study protocol was presented to the institutional protocol committee, and ethical approval was obtained from the Institutional Ethics Committee of Krishna Vishwa Vidyapeeth, Karad, Maharashtra, India (approval number: KVV/IEC/10/2025; Protocol No. 300/2025-2026). A quasi-experimental pre-post interventional study was undertaken at the Department of Paediatric Physiotherapy, Krishna College of Physiotherapy, Krishna Vishwa Vidyapeeth (Deemed to be University) and at Chaitanya Bal Rugnalaya, Karad, Maharashtra, India. Before recruitment, consent was received from the parents of all participants, and assent was obtained from children aged five years and older to ensure voluntary participation. 

Participants

Based on feasibility and pilot study considerations, a total of 15 participants meeting the eligibility criteria were recruited. A convenient sampling method was utilized for participant recruitment. The study included 15 children aged between five and 12 years, with a mean age of 8.27 ± 2.31 years. The sample consisted of nine male (60.0%) and six female (40.0%) patients, indicating a predominantly male distribution. Participants were recruited through convenience sampling and categorised under the Gross Motor Function Classification System (GMFCS) [12] Levels I and III. The largest age subgroup was eight to nine years (40.0%), followed by four to five years (20.0%), six to seven years (20.0%), and 10 to 12 years (20.0%), reflecting the heterogeneous features of cerebral palsy with genu recurvatum among children. 

Inclusion criteria

Children having spastic diplegic cerebral palsy classified under GMFCS Levels I and III, aged between five and 12 years, and presenting with genu recurvatum were enrolled in the study. It is acknowledged that combining GMFCS Levels I and III may introduce heterogeneity in baseline motor function; however, given the small sample size, stratified analyses by GMFCS level were not feasible, and the findings should therefore be interpreted with consideration of this variability. Participants were required to be free from acute illness, uncontrolled seizures, or recent surgical procedures and able to follow simple verbal instructions during intervention and assessment procedures.

Exclusion criteria

Children presenting with severe cognitive deficits, uncontrolled seizures, recent orthopaedic surgery or botulinum toxin injection within the last six months, severe visual or auditory deficits, or other associated neurological or musculoskeletal impairments that could hinder participation were excluded from the study.

Outcome measures

The subsequent outcome measures were employed to evaluate the effectiveness of pulley therapy combined with vibration therapy on spasticity, gross motor function, and genu recurvatum in children with spastic cerebral palsy.

The GMFM-88 [13] was utilised to evaluate gross motor skills in five areas: sitting, standing, crawling and kneeling, lying and rolling, walking, running, and leaping. The GMFM-88, a validated and standardised assessment instrument created especially for children with cerebral palsy, is frequently used to assess improvements in functional motor performance. Future studies may consider supplementing GMFM-88 with functional independence measures such as the Pediatric Evaluation of Disability Inventory (PEDI) or WeeFIM, particularly in children aged five years and above with GMFCS Levels I and III, to provide a more comprehensive evaluation of functional outcomes beyond gross motor performance.

The Modified Ashworth Scale (MAS) [14] was employed to evaluate lower limb spasticity. The MAS evaluates the degree of resistance during passive movement and grades the severity of muscle hypertonicity. It demonstrates good inter-rater reliability and is commonly used in paediatric neurological rehabilitation settings. It is acknowledged that the MAS is a subjective measure and does not specify the angle at which spasticity occurs; the Tardieu Scale, which captures velocity-dependent spasticity and identifies the angle of catch, would provide a more precise assessment and is recommended for use in future studies. 

Goniometric measurement was applied to measure the degree of genu recurvatum (knee hyperextension). A universal goniometer was utilized to objectively measure the angle of knee hyperextension prior to and following the intervention. This assessment provided quantitative information regarding knee alignment and improvement in joint positioning.

Prior to initiation of the intervention, comprehensive pre-assessment evaluations were conducted to establish baseline measures of the dependent variables. Pre-assessment included the following: 1. Assessment of the gross motor function using the GMFM-88 scale; 2. Evaluation of spasticity with MAS for lower limb muscles; 3. Measurement of genu recurvatum using goniometric assessment of knee hyperextension; 4. Collection of baseline subject characteristics such as age and gender, type of cerebral palsy, GMFCS level, and degree of genu recurvatum prior to intervention. These baseline findings were used for comparison with post-intervention outcomes to determine the effect of the treatment protocol. 

Intervention protocol 

The intervention procedure was conducted over 12 weeks with four sessions/week. Each session lasted approximately 45-50 minutes, including rest periods. The intervention protocol was designed to improve gross motor skills, reduce spasticity, and correct genu recurvatum in children with spastic cerebral palsy through a combination of pulley therapy, vibration therapy, stretching, and functional training exercises. The intervention session began with gentle warm-up and stretching exercises for approximately five to 10 minutes to prepare the muscles and joints for activity. Stretching was focused on the quadriceps, hamstrings, gastrocnemius, and soleus muscles to reduce muscle tightness, improve flexibility, and prevent contracture formation. Vibration therapy was administered using the Proton™ Vibration Plate Exercise Machine (Proton Healthcare, Bengaluru, India), a vibration platform with 99 speed levels and a frequency range of 20-30 Hz. Participants performed the specified positions on the device as outlined across the three phases of the intervention protocol. The detailed intervention protocol followed during Phase I (Weeks 1-4), which included low-complexity activities and initial motor training progression, is presented in Table 1.

**Table 1:** Phase I (Weeks 1–4), low-complexity activities and initial motor training progression Reps: Repetitions; MVC: Maximum Voluntary Contraction; RPE: Rating of Perceived Exertion References: [5,15,16]

Name of Exercise	Frequency	Intensity
PHASE 1 (Weeks 1-4)		
Pulley Exercise		
1. Hamstring Curls (Supine)	2 Sets x 15 Reps	30-40% MVC/RPE 10-11 (light effort)
2. Tibialis Anterior Activation	2 Sets x 15 Reps	30-40% MVC/RPE 10-11 (light effort)
VIBRATION THERAPY (Proton™ Vibration Plate Exercise Machine)		
Semi-squat Position (20 Degrees)	20-25 Hz (Proton™ Vibration Plate Exercise Machine)	30-40% MVC/RPE 10-11 (light effort)
STRETCHING EXERCISES		
1. Passive Quadriceps Stretch	30 secs x 3 Reps/Leg	30-40% MVC/RPE 10-11 (light effort)
2. Gastrocnemius & Soleus Stretch	30 secs x 3 Reps/Leg	30-40% MVC/RPE 10-11 (light effort)
FUNCTIONAL ACTIVITIES		
1. Sit to Stand from Therapy Ball	1 Set x10 Reps	30-40% MVC/RPE 10-11 (light effort)
2. Step-Ups on Low Step	1 Set x10 Reps	30-40% MVC/RPE 10-11 (light effort)

Moderate-complexity progression protocol implemented during Phase II (Weeks 5-8), including advancement of coordination, balance, and functional training activities, is displayed in Table 2. 

**Table 2:** Phase II (Weeks 5–8), advancement of coordination, balance, and functional training activities Reps: Repetitions; MVC: Maximum Voluntary Contraction; RPE: Rating of Perceived Exertion References: [8,15,17]

Name of Exercise	Frequency	Intensity
PHASE 2 (Weeks 5-8)		
PULLEY EXERCISE		
1. Resisted Hip & Knee Flexion-Extension	3 Sets x 12 Reps	40-50% MVC/RPE 12-13 (moderate effort)
2. Hamstring Curls in Prone Position	3 Sets x 12 Reps	40-50% MVC/RPE 12-13 (moderate effort)
3. Toe Dorsiflexion with Pulley	3 Sets x 12 Reps	40-50% MVC/RPE 12-13 (moderate effort)
VIBRATION THERAPY (Proton™ Vibration Plate Exercise Machine)		
Semi-squat Position (20-30 Degrees)	25-30 Hz (Proton™ Vibration Plate Exercise Machine)	40-50% MVC/RPE 12-13 (moderate effort)
STRETCHING EXERCISES		
1. Passive Quadriceps Stretch	30 secs x 3 Reps/Leg	40-50% MVC/RPE 12-13 (moderate effort)
2. Gastrocnemius & Soleus Stretch	30 secs x 3 Reps/Leg	40-50% MVC/RPE 12-13 (moderate effort)
FUNCTIONAL ACTIVITIES		
1. Mini Squats on Platform	1 Set x 10 Reps	40-50% MVC/RPE 12-13 (moderate effort)
2. Balance Board	1 Set x 10 Reps	40-50% MVC/RPE 12-13 (moderate effort)
3. Step-Hold Task	1 Set x 10 Reps	40-50% MVC/RPE 12-13 (moderate effort)
4. Mirror Feedback Walking	1 Set x 10 Reps	40-50% MVC/RPE 12-13 (moderate effort)

The high-complexity intervention progression followed during Phase III (Weeks 9-12), including advanced task-oriented and dynamic motor activities, is presented in Table 3.

**Table 3:** Phase III (Weeks 9–12), advanced task-oriented and dynamic motor activities Reps: Repetitions; MVC: Maximum Voluntary Contraction; RPE: Rating of Perceived Exertion References: [16,18,19]

Name of Exercise	Frequency	Intensity
PHASE 3 (Weeks 9-12)		
PULLEY EXERCISE		
1. Unilateral Resisted Knee Flexion-Extension	3 Sets x 10 Reps/Leg	50-60% MVC/RPE 13-14 (moderate-hard effort)
2. Pulley-based core activation with Leg Lifts.	3 Sets x 10 Reps/Leg	50-60% MVC/RPE 13-14 (moderate-hard effort)
3. Supine Marching with Pulley	3 Sets x 10 Reps/Leg	50-60% MVC/RPE 13-14 (moderate-hard effort)
VIBRATION THERAPY (Proton™ Vibration Plate Exercise Machine)		
Stance Tandem (20-30 Degrees)	25-30 Hz (Proton™ Vibration Plate Exercise Machine)	50-60% MVC/RPE 13-14 (moderate-hard effort)
STRETCHING EXERCISES		
1. Passive Quadriceps Stretch	30 secs x 3 Reps/Leg	50-60% MVC/RPE 13-14 (moderate-hard effort)
2. Gastrocnemius & Soleus Stretch	30 secs x 3 Reps/Leg	50-60% MVC/RPE 13-14 (moderate-hard effort)
FUNCTIONAL ACTIVITIES		
1. Toss Ball Catch-Catch	1 Set x 10 Reps	50-60% MVC/RPE 13-14 (moderate-hard effort)
2. Reach-Outs in Different Directions	1 Set x 10 Reps	50-60% MVC/RPE 13-14 (moderate-hard effort)
3. Sit to Stand with Weight Shift	1 Set x 10 Reps	50-60% MVC/RPE 13-14 (moderate-hard effort)
4. Step-Ups with Contralateral Foot Control	1 Set x 10 Reps	50-60% MVC/RPE 13-14 (moderate-hard effort)

Statistical analysis

Statistical analysis was performed using IBM SPSS Statistics software, version 25.0 (IBM Corp., Armonk, NY, USA). Demographic characteristics, including age and gender, were analyzed using descriptive statistics and presented as frequencies, percentages, mean, and standard deviation (SD).

Data normality was assessed using the Shapiro-Wilk test. Outcome measures included the GMFM-88, MAS, and goniometric measurement of genu recurvatum. Pre- and post-intervention values were compared using the paired samples t-test.

Mean and SD were calculated for all outcome measures. Effect size was estimated using Cohen's d to determine the magnitude of treatment effects. Statistical significance was set at p < 0.05.

Microsoft Excel (Microsoft Corp., Redmond, WA, USA) was used for data tabulation and preparation of tables, graphs, and charts for visual presentation of the study findings.

## Results

The pre- and post-intervention comparison demonstrated significant improvements in gross motor function, spasticity, and genu recurvatum following the 12-week intervention program. Table 4 presents the pre- and post-intervention GMFM-88 scores of the participants along with the statistical analysis. 

**Table 4:** Comparison of pre- and post-intervention values of Gross Motor Function Measure-88 (GMFM-88) using the paired t-test (n = 15)

Scale	Pre-intervention value	Post-intervention value	t value	P-value	Cohen’s d
GMFM-88 (n=15)	0.690 ±0.05	0.762 ±0.06	6.01	< 0.001	1.55

Table 4 demonstrates a significant improvement in GMFM-88 scores following combined pulley therapy and vibration therapy, with scores increasing from 0.690 ± 0.05 to 0.762 ± 0.06 (t = 6.01; p < 0.001). These results indicate the combined intervention was associated with enhancements in gross motor function, including movement control, postural stability, and functional independence in children presenting with spastic cerebral palsy.

Table 5 shows the pre- and post-intervention MAS scores and their statistical comparison.

**Table 5:** Comparison of pre- and post-intervention values of Modified Ashworth scale using the paired t-test (n = 15)

Scale	Pre-intervention value	Post-intervention value	t value	P-value	Cohen’s d
Modified Ashworth Scale (n=15)	1.90 ± 0.38	1.40 ± 0.35	7.2	< 0.001	1.86

Table 5 demonstrates a significant reduction in spasticity following the combined intervention, with MAS scores decreasing from 1.90 ± 0.38 to 1.40 ± 0.35 (t = 7.2; p < 0.001). The clinically meaningful reduction in tone may contribute to improved ease of movement and reduced risk of secondary musculoskeletal complications in children with spastic cerebral palsy. 

Table 6 shows the comparison of pre- and post-intervention goniometric measurements, highlighting the changes in range of motion after the intervention

**Table 6:** Comparison of pre- and post-intervention goniometric measurements using the paired t-test

Scale	Pre-intervention value	Post-intervention value	t value	P-value	Cohen’s d
Goniometric measurements (n=15)	11.00° ± 1.8	7.8° ± 1.5	8.6	< 0.001	2.22

Table 6 demonstrates a statistically significant improvement in joint range of motion following the combined intervention, with goniometric measurements decreasing from 11.00° ± 1.8 to 7.8° ± 1.5 (t = 8.6; p < 0.001; Cohen's d = 2.22), indicating a large effect size.

## Discussion

The present study evaluated the effectiveness of pulley therapy combined with vibration therapy on spasticity, gross motor function, and genu recurvatum in children with spastic cerebral palsy. Following the 12-week intervention program, significant improvements were observed across all outcome measures, indicating the potential effectiveness of the combined intervention in improving motor function and reducing spasticity.

The improvement in GMFM-88 scores observed in the present study is consistent with previous rehabilitation studies conducted in children with cerebral palsy. Pyrzanowska et al. [20] reported that combined intensive rehabilitation approaches improve gait performance and functional mobility in children with cerebral palsy. Similarly, Zardo et al. [21] demonstrated significant improvements in gross motor function following a multimodal neurorehabilitation program. Sinha et al. [22] also emphasized the importance of structured and goal-oriented rehabilitation in improving functional motor outcomes. These findings support the results of the present study and suggest that multimodal rehabilitation programs can positively influence motor performance in children with cerebral palsy.

The significant improvement in gross motor function may be attributed to the combined therapeutic mechanisms of pulley therapy and vibration therapy. Pulley therapy facilitates task-specific strengthening, active participation, selective motor control, and functional movement training. Vibration therapy enhances proprioceptive input, neuromuscular activation, and sensory feedback. Together, these interventions may promote motor learning, improve movement coordination, and enhance functional independence.

A significant reduction in MAS scores was observed following the intervention, indicating reduced lower-limb spasticity. Vibration therapy is believed to influence spinal reflex pathways through stimulation of Ia afferent fibers, thereby facilitating presynaptic and reciprocal inhibition and reducing alpha motor neuron excitability [15]. Calabrò et al. [18] highlighted the importance of repetitive task-specific rehabilitation in promoting neuroplastic adaptations and motor recovery. Furthermore, Ahlborg et al. [17] demonstrated beneficial effects of whole-body vibration on spasticity, muscle strength, and motor performance in individuals with cerebral palsy. Opheim et al. [19] also reported improvements in gross motor function following repeated intensive rehabilitation interventions, supporting the role of sustained rehabilitation in improving functional outcomes.

One of the most clinically important findings of the present study was the reduction in genu recurvatum. The present study objectively demonstrated a reduction in knee hyperextension angle following the intervention; however, the underlying mechanisms responsible for this change were not directly measured. It is speculated that improvements in knee alignment may potentially be related to enhanced hamstring strength, improved tibialis anterior activation, and better neuromuscular coordination, though this remains unconfirmed, as muscle strength, electromyography activity, and gait kinematics were not assessed in the current study. Future studies should incorporate these outcome measures to elucidate the mechanisms underlying changes in genu recurvatum, better postural control, and improved neuromuscular coordination achieved through progressive pulley-based strengthening exercises. The stretching component of the intervention may have contributed to improved flexibility and reduced muscle tightness. Qamar et al. [5] reported that structured stretching interventions can effectively reduce spasticity and improve joint mobility in children with cerebral palsy, supporting the findings of the present study.

The vibration parameters utilized in the present intervention are supported by the findings of Ritzmann et al. [15], who concluded that vibration therapy may improve motor function and reduce spasticity in individuals with cerebral palsy. In addition, Willoughby et al. [16] reported that progressive weight-bearing and treadmill-based rehabilitation programs improve walking ability and functional mobility in children with cerebral palsy. Similar principles of progressive loading and functional training were incorporated into the present rehabilitation protocol.

Clinical implications

The findings of the present study suggest that combining pulley therapy with vibration therapy may represent a practical, cost-effective, and clinically feasible rehabilitation strategy for children with spastic cerebral palsy presenting with genu recurvatum. However, given the absence of a control group and the preliminary nature of the proposed therapeutic mechanisms, these findings should be interpreted with caution and require confirmation through future randomized controlled trials before definitive clinical recommendations can be made. Pending further validation, the combined intervention protocol may be considered for incorporation into routine pediatric neurorehabilitation programs with the potential to improve gross motor performance, reduce lower-limb spasticity, and enhance knee alignment and functional mobility in this population.

Limitations

The present study has several limitations. The sample size was relatively small, and participants were recruited using convenience sampling. The absence of a control group represents a significant methodological limitation, as it precludes causal attribution of the observed improvements solely to the combined pulley and vibration therapy intervention. Confounding factors such as natural maturation, concurrent standard physiotherapy, therapist attention effects, and repeated testing cannot be ruled out as contributing to the observed gains. Long-term follow-up was not conducted to determine the sustainability of treatment effects. Therefore, the findings should be interpreted with caution and may not be generalized to the wider cerebral palsy population.

The inclusion of children across GMFCS Levels I and III introduced functional heterogeneity within the sample. Children classified under GMFCS Level I demonstrated near-normal functional ability with minimal spasticity-related restrictions, which may have influenced the overall treatment response. Future studies should consider stratified analysis by GMFCS level to better understand differential treatment effects across functional subgroups. 

As all participants received standard physiotherapy, pulley therapy, and vibration therapy simultaneously, the independent therapeutic contribution of each component cannot be determined. The observed improvements should therefore be attributed to the combined protocol as a whole rather than to any single intervention component.

Although MAS is an ordinal scale, parametric analysis was performed because the data satisfied assumptions of normality according to the Shapiro-Wilk test. This approach has been reported in previous rehabilitation studies; however, future studies may consider non-parametric analyses.

Future recommendations

Future randomized controlled trials with larger sample sizes, longer follow-up periods, and comparison groups are recommended to further investigate the effectiveness of pulley therapy combined with vibration therapy in children with cerebral palsy and genu recurvatum.

## Conclusions

The present study found that pulley therapy combined with vibration therapy was associated with statistically significant improvements in spasticity, gross motor function, and genu recurvatum in children with spastic cerebral palsy following a structured 12-week intervention program. These findings suggest preliminary promise for this combined rehabilitation approach in pediatric neurorehabilitation. However, given the quasi-experimental design, absence of a control group, convenience sampling, and small sample size, the findings should be interpreted with caution. Furthermore, as all participants received standard physiotherapy concurrently, the independent therapeutic contribution of each intervention component cannot be determined from the current study design.

Future randomized controlled trials with larger sample sizes, comparison groups, and separate intervention arms are warranted to confirm the effectiveness of this combined protocol and to isolate the specific effects of pulley therapy and vibration therapy individually in children with spastic cerebral palsy and genu recurvatum.
